# Weight Loss and Melatonin Reduce Obesity-Induced Oxidative Damage in Rat Testis

**DOI:** 10.1155/2013/836121

**Published:** 2013-09-08

**Authors:** Dogan Atilgan, Bekir S. Parlaktas, Nihat Uluocak, Fikret Erdemir, Sahin Kilic, Unal Erkorkmaz, Huseyin Ozyurt, Fatma Markoc

**Affiliations:** ^1^Gaziosmanpasa University, Faculty of Medicine, Department of Urology, 60100 Tokat, Turkey; ^2^Sakarya University, Faculty of Medicine, Department of Biostatistics and Medical informatics, 54100 Sakarya, Turkey; ^3^Gaziosmanpasa University, Faculty of Medicine, Department of Biochemistry, 60100 Tokat, Turkey; ^4^Gaziosmanpasa University, Faculty of Medicine, Department of Pathology, 60100 Tokat, Turkey

## Abstract

*Aim*. We aimed to evaluate the antioxidant effects of weight loss and melatonin on the obesity-induced oxidative damage in rat testes. *Materials and Methods*. 28 male Wistar albino rats were randomly divided into 4 groups, each consisting of 7 rats: control group (Group 1), obesity group (Group 2), obesity + MLT group (Group 3), and weight loss group (Group 4). Rats were weighed at the beginning and at the end of the study. Bilateral orchiectomy was performed and 5 cc blood samples were obtained from all of the rats. Superoxide dismutase (SOD), malondialdehyde (MDA), and protein carbonyl (PC) levels were analysed in the testicular tissues and serum. Spermatogenesis was evaluated with the Johnsen scoring system. *Results*. The testicular tissue and serum levels of MDA, PC, and SOD activity were increased in the obesity group in comparison to the sham operated group (*P* < 0.05). Weight loss and melatonin treatment ameliorated MDA, PC, and SOD levels in testicular tissue and serum significantly (*P* < 0.05). There was no significant difference between groups in terms of mean Johnsen score (*P* = 0.727). *Conclusion*. Experimentally created obesity caused oxidative stress and both melatonin and weight loss reduced oxidative stress parameters in rat testes.

## 1. Introduction

Obesity is the accumulation of excessive fat in adipose tissue and it is one of the most important health problem in the world at the present time which affects both gender and all age groups [1]. According to WHO (World Health Organisation), overweight and obesity are major risk factors for a number of chronic diseases including diabetes, cardiovascular diseases, cancer, and certain reproductive and metabolic disorders. Once considered as a problem only in high income countries, overweight and obesity are now dramatically on the rise in low- and middle-income countries, particularly in urban settings [2].

The relevance of increased BMI with poor semen quality [3], decreased sperm concentration [4–7], decreased normal-motile sperm cells, and increased DNA fragmentation index [8, 9] was shown in many studies recently. In contrast to these results, there are also some other studies that claimed no relationship between obesity and sperm concentration, motility or morphology, as well as [10–12]. The mechanisms that describe the relationship between obesity and male infertility are still unclear. Increased DNA fragmentation [9], oxidative stress (OS) [13], and hormonal imbalance [14, 15] have been proposed as the likely mechanisms of sperm abnormalities associated with obesity. 

Oxidative injury is a complex phenomenon that causes destruction in both local and remote tissues. Oxidative injury develops when there is excessive production of reactive oxygen species (ROS) and/or free radicals, which exceeds the natural antioxidant defence mechanisms in the body. The OS can produce important destructive effects in tissues by causing alterations in the cell membranes leading to irreversible cellular damage. Eventually, oxidative damage causes an increase in tissue levels of malondialdehyde (MDA) and protein carbonyl (PC), which are the end products of lipid peroxidation and protein oxidation, respectively, [16]. Many studies have shown that obesity induced OS and this phenomenon led to tissue damage [17, 18]. 

As the chief secretory product of the pineal gland, melatonin (MLT) (N-acetyl-5-methoxytryptamine) functions as a synchronizer of the biological clock and has powerful antioxidant activity [19]. The protective role of melatonin by reducing OS and lipid peroxidation has been reported in various experimental models. In this study, we aimed to show the antioxidant effects of weight loss in comparison with melatonin which is known as a potent antioxidant agent on the obesity-induced oxidative damage in rat testes.

## 2. Materials and Methods

After obtaining local ethical committee permission, a total of 28 male 5-6 months old Wistar albino rats were used in the study. The experimental animals were housed at 18–22°C, under a 12 h light/12 h dark throughout the study. All surgical procedures were performed under xylazine/ketamine anesthesia in sterile conditions. All rats were sacrificed after the experimental procedures.

The rats were randomly divided into four groups each consisting of 7 rats. Group 1 (sham-operated control group, *n* = 7) underwent a sham operation to determine basal values for biochemical and histological evaluation. Bilateral orchiectomy was performed out through midline abdominal incision. Group 2 (obesity group, *n* = 7) was designed to create obesity-induced oxidative damage. In this group rats were fed with high calorie and fat-containing diet for a period of 8 weeks. After 8 weeks, bilateral orchiectomy was performed. Group 3 (obesity + MLT group, *n* = 7) was designed to determine the effect of melatonin on antioxidant parameters in the obese rats. In this group rats, were also fed with high calorie and fat-containing diet for same period. After 8 weeks of experimental period, melatonin (50 mg/kg, i.p) was administered in a single dose 30 min prior to surgical procedure and bilateral orchiectomy was performed. Group 4 (weight loss group, *n* = 7) was designed to show effects of weight loss on the oxidative parameters in the obese rats. After 8 weeks of period with high calorie and fat-containing diet, rats were fed with standard pellet diet for an additional period of 4 weeks. After 12 weeks, bilateral orchiectomy was performed. 

All of the rats were weighed at the beginning and at the end of the study and the data were recorded. The testes of each rat were fixed in 10% formaldehyde solution and were stored at −70°C pending biochemical studies. Approximately 5 cc blood samples were obtained from the vena cava inferior of all the rats. Biochemically antioxidant enzyme activities (SOD), malondialdehyde (MDA), protein carbonyl (PC) levels in testicular tissue and serum were determined. Testicular tissues were also examined histopathologically and spermatogenesis was evaluated with Johnsen's scoring system.

*Histological Criteria for the Modified Johnsen Scoring Are as Follows:*

  full spermatogenesis (Score 10),
  slightly impaired spermatogenesis, many late spermatids, disorganized epithelium (Score 9),
  less than five spermatozoa per tubule, few late spermatids (Score 8),
  no spermatozoa, no late spermatids, many early spermatids (Score 7),
  no spermatozoa, no late spermatids, few early spermatids (Score 6),
  no spermatozoa or spermatids, many spermatocytes (Score 5),
  no spermatozoa or spermatids, few spermatocytes (Score 4),
  spermatogonia only (Score 3),
  no germinal cells, Sertoli cells only (Score 2),
  no seminiferous epithelium (Score 1).

### 2.1. Biochemical Analysis

The testicular tissues were homogenized in five volumes of ice-cold tris-HCl buffer (50 mM, pH 7.4) containing 0.50 mL/l Triton x-100. Then homogenization procedure (homogenizer: IKA Ultra-Turrax T 25 Basic, Germany) was carried out for 2 min at 13,000 rpm. All procedures were performed at 4°C. The homogenate, supernatant, and extracted samples were prepared and the following determinations were made on the samples using commercial chemicals supplied by Sigma (St. Louis, USA). Protein measurements were made in the samples according to the method explained elsewhere. 

#### 2.1.1. Determination of Tissue Superoxide Dismutase (SOD) Activity

Total (Cu-Zn and Mn) SOD (EC 1.15.1.1) activity was determined according to the method of Sun et al. [20]. The principle of the method was based on inhibition of nitroblue tetrazolium (NBT) reduction by the xanthine-xanthine oxidase system as a superoxide generator. Activity was assessed in the ethanol phase of the supernatant after 1.0 mL of ethanol-chloroform mixture (5/3, v/v) was added to the same volume of sample and centrifuged. One unit of SOD was defined as the amount causing 50% inhibition in the NBT reduction rate. The SOD activity was expressed as U/mg protein for testicular tissue.

#### 2.1.2. Determination of Malondialdehyde (MDA) Level

The tissue thiobarbituric acid-reactive substance level was determined by a method based on reaction with thiobarbituric acid (TBA) at 90–100°C [21]. In the TBA test reaction, MDA or MDA-like substances and TBA react to produce a pink pigment with an absorption maximum at 532 nm. The reaction was performed at 2-3 pH and 90°C for 15 min. The sample was mixed with two volumes of cold 10% (w/v) trichloroacetic acid to precipitate the protein. The precipitate was pelleted by centrifugation, and an aliquot of the supernatant was reacted with an equal volume of 0.67% (w/v) TBA in a boiling water bath for 10 min. After cooling, the absorbance was read at 532 nm. Results were expressed as nmol/g wet tissue for testicular tissue according to the standard graphics prepared from measurements with a standard solution (1,1, 3,3-tetramethoxypropane).

#### 2.1.3. Determination of Tissue Protein Carbonyl (PC)

The carbonyl contents were determined spectrophotometrically (Cintra 10 E, Austria) based on reaction of carbonyl group with 2,4-dinitrophenylhydrazine to form 2,4 dinitrophenylhydrazone [22]. The results were given as nmol/mL for testicular tissue.

### 2.2. Histopathological Examination

The testes were fixed in 10% formaldehyde solution. The tissues were processed for paraffin embedding and 5 *μ*m thick sections were stained with hematoxylin and eosin. The sections were analyzed for the general architecture, spermatogenesis, the basement membrane of seminiferous tubules, and the presence or absence of leydig cell hyperplasia. The seminiferous tubules were graded according to the Johnsen scoring system. Twenty tubular sections in each testis were evaluated and mean Johnsen score was calculated. The normal mean Johnsen score was accepted as >9.39.

### 2.3. Statistical Analysis

Kruskall-Wallis test was used to compare the weights, biochemical, and histopathological parameters among groups. When Kruskall-Wallis test results were significant, Bonferroni adjusted Mann-Whitney *U* test was used in the paired comparisons. The continuous variables were presented as the mean ± standard deviation and range (min-max values). Wilcoxon rank sum test was used to compare the weights of rats between measures of base and 8th week. Friedman test was used to compare the weights of rats among measures of base and 8th and 12th weeks. When Friedman test result was significant, Bonferroni adjusted Wilcoxon rank sum test was used in the paired comparison. The weights were presented as the mean ± standard deviation. A *P* value <0.05 was considered significant. Analyses were performed using commercial software (IBM SPSS Statistics 20, SPSS Inc., an IBM Co., Somers, NY, USA).

## 3. Results

The weights of rats at the beginning and at the end of study are presented in Table 1. Mean weight gains after 8 weeks of high calorie diet were 12.3 gr/rat, 68.6 gr/rat, 71.4 gr/rat, and 72.9 gr/rat for each group, respectively. Mean weight loss in Group 4 was 28.2 gr/rat after additional 4 weeks of normal pellet diet. Mean initial weight of the rats in Group 3 (obesity + MLT) was statistically significant in comparison to Group 1 (*P* < 0.05). In obesity, obesity + MLT, and weight loss groups, the mean weight after 8 weeks of high calorie diet intake and the weight differences were statistically significant in comparison with the control group (*P* < 0.05). In addition, in Group 4, the weight after additional 4 weeks of normal pellet diet intake was statistically significant in comparison with mean initial weight of this group (*P* < 0.05).

The results and the analysis of testicular tissue MDA, SOD and PC values in all groups are presented in Table 2. The testicular tissue levels of MDA and PC were increased in the obesity group in comparison to the sham-operated group (*P* < 0.05). Weight loss and melatonin treatment ameliorated MDA and PC levels in testicular tissues significantly (*P* < 0.05). SOD activity was increased in the obesity group. Weight loss and melatonin treatment caused decreased SOD activity in comparison to obesity group (*P* < 0.05). 

The results of serum MDA, SOD, and PC values in all groups are presented in Table 3. The levels of serum MDA and PC were increased in the obesity group in comparison to sham-operated group (*P* < 0.05). Weight loss and melatonin treatment reduced MDA and PC levels in serum significantly (*P* < 0.05). Similarly, antioxidant enzyme activity (SOD) was increased in the obesity group. Weight-loss and melatonin treatment reduced SOD activity in comparison with the obesity group (*P* < 0.05). 

In histopathological examination of the testicular tissues, there was no statistically significant difference between all of the groups in terms of mean Johnsen score (Table 4). However, melatonin treatment and weight loss caused nonsignificant improvement in mean Johnsen score in comparision to obesity group.

## 4. Discussion

Obesity is considered as a major health problem that has become an epidemic disease throughout the world. Although the association of obesity with various systemic diseases is well known, the relationship between obesity and male reproductive disorders is still obscure. Therefore, many studies have been carried out to clarify this relationship in recent years [23, 24]. 

Development of a systemic OS due to obesity has been shown by both experimental and clinical studies. Weisberg et al. [25] and Xu et al. [26] indicated that infiltration of macrophages, which is the important source of inflammatory cytokines, is the major reason of the ROS production in the adipose tissue. In another study, Furukawa et al. reported that in nondiabetic human subjects, fat accumulation was closely correlated with the markers of systemic OS. They also concluded that increased OS in accumulated fat led to dysregulated production of adipocytokines and selective increase in ROS production in adipose tissue. Hence, increased ROS secretion into the peripheral blood from accumulated fat may be the possible cause of systemic OS in obesity [27]. 

The ROS are generated by mitochondrial cytochrome oxidase, nicotinamide adenine dinucleotide phosphate oxidase, xanthine oxidase, lipoxygenase, cyclooxygenase, heme oxygenase, cytochrome P-450 enzymes, nitric oxide synthase (NOS), and various other oxidase enzymes. Although the ROS are essential for normal reproductive functions, namely, sperm capacitation and acrosome reaction, in physiological levels, they lead to adverse effects at higher concentrations [28–30]. The spermatids and mature spermatozoa are quite sensitive to ROS because their membranes are rich in polyunsaturated fats [31]. Hence, sperm motility and morphology may be impaired and sperm cell death may occur depending on increased ROS levels in OS conditions [32]. Membrane lipid peroxidation may generate reactive carbonyl compounds such as MDA, which is one of the most reliable indicators of ROS-induced tissue damage [21]. In addition, PC level which results from oxidative damage of protein structures or direct oxidation of amino acids is considered as an indicator of DNA fragmentation [22]. In this study, we observed that high-fat diet was effective in promoting obesity as demonstrated by higher body weights and obesity induced elevations on ROS levels both in systemic circulation and testicular tissues. The levels of antioxidant enzymes were elevated to compensate the ROS and this was an indirect indicator of increased ROS levels. Elevated SOD, PC, and MDA levels in blood and testicular tissues of obese rats compared to controls (*P* < 0.05) showed that obesity constituted a systemic OS and this condition appeared in testicular tissue and serum. 

In terms of mean Johnsen score, there was no statistically significant difference between obesity, obesity + MLT, and weight loss groups. However, the mean Johnsen scores of obesity + MLT and weight loss groups were closer to the control group compared with obesity group. Although there was no statistically significant difference between the groups concerning Johnsen scores, it may be speculated that the impaired Johnsen scores seen in obesity group may be the result of distorted membrane structure and increased DNA fragmentation of sperm cells due to OS.

Depending on its antioxidant properties, MLT helps antioxidant enzymes to clean ROS. As a result of this in obesity + MLT group, SOD, MDA, and PC levels decreased to a level closer to the sham group. These results may arise a thought that melatonin has beneficial effects on inflammation that reduces neuromediators migration due to OS.

There are a limited number of studies in the literature that showed beneficial effects of weight loss on the OS and sperm parameters due to obesity. Dandona et al. demonstrated that key indexes of lipid peroxidation (TBARS, 9-HODE, and 13-HODE) as well as those of oxidative damage to proteins and amino acids (carbonylated proteins, *o*-tyrosine, and *m*-tyrosine) were significantly higher in the obese than in normal subjects and that they were decreased significantly after dietary restriction and weight loss. They also showed that the levels of ROS have been decreased markedly in association with dietary restriction and weight loss after the institution of a 1000-calorie diet and this effect was evident at 1 week and persisted over 4 weeks of diet [33]. Additionally, Mohn et al. concluded that prepubertal severely obese children have been presented with a highly altered oxidant/antioxidant status, which was completely reversible with dietary restriction and weight loss [34]. In a cohort study, Håkonsen et al. had indicated that the altered androgen profile tended to improve following weight loss and that weight loss potentially led to improvement in semen quality [35]. In this study, we found that in weight loss group SOD, MDA, and PC levels were significantly decreased compared to obesity group and there was no significant difference between control, weight loss and melatonin treated groups. 

In conclusion, obesity induced OS in both testicular tissues and systemic circulation but it did not affect the sperm morphology significantly. One dose injection of potent antioxidant agent MLT and weight loss reduced OS parameters and improved the sperm morphology slightly. 

## Figures and Tables

**Table 1:** Weights of rats at the beginning and at the end of study.

	Group 1	Group 2	Group 3	Group 4	*P
Weight (base)	311.43 ± 2.94	314.29 ± 3.59	320 ± 3.65	315.71 ± 3.40	0.003^a^
Weight (8th week)	323.71 ± 1.80	383.93 ± 5.20	391.43 ± 4.35	388.57 ± 5.03	<0.001^b^
**P	**0.017**	**0.018**	**0.018**	**0.018**	
Weight differences (base to 8th week)	12.29 ± 1.89	69.64 ± 6.73	71.43 ± 7.59	72.86 ± 6.01	0.001^b^
Weight (12th week)	—	—	—	369.43 ± 5.56	
***P	—	—	—	0.001^c^	

Data were shown as mean ± standard deviation.

*P values of the comparisons among four groups.

**P values of the comparisons between base and 8th week weights separately for groups.

***P values of the comparisons among base 8th week and 12th week weights for group 4.

^a^There was statistically significant difference between group 1 and group 3. However there were no statistically significant difference between other pairwise comparisons.

^b^There was statistically significant difference between group 1 and other groups. However there were no statistically significant difference between other pairwise comparisons.

^c^There was statistically significant difference between measures of base and 8th and 12th week. However there were no statistically significant difference between measures of 8th and 12th week.

**Table 2:** Tissue MDA, PC levels and SOD activities.

	Group 1 (*n* = 7)	Group 2 (*n* = 7)	Group 3 (*n* = 7)	Group 4 (*n* = 7)	*P*
SOD (U/mg protein)	0.008 ± 0.002 (0.006–0.011)	0.035 ± 0.009 (0.018–0.044)^a^	0.014 ± 0.007 (0.007–0.026)^b^	0.014 ± 0.002 (0.011–0.016)^b^	**0.001**
MDA (nmol/g wet tissue)	20.10 ± 11.69 (9.31–40.2)	42.36 ± 12.28 (28.46–61.52)^a^	23.65 ± 6.46 (15.27–32.64)^b^	21.05 ± 15.33 (12.28–51.88)^b^	**0.017**
Protein Carbonyl (nmol/mg prot)	2.90 ± 0.70 (1.69–3.84)	4.59 ± 0.78 (3.49–5.48)^a^	3.66 ± 0.84 (2.68–4.89)^b^	3.55 ± 0.95 (2.16–4.78)^b^	**0.038**

Data were shown as mean ± standard deviation and range (min–max).

^a^There was statistically significant difference from group 1.

^b^There was statistically significant difference from group 2.

**Table 3:** Serum SOD, MDA, and PC levels of all groups.

	Group 1 (*n* = 7)	Group 2 (*n* = 7)	Group 3 (*n* = 7)	Group 4 (*n* = 7)	*P*
SOD (U/mg protein)	1.93 ± 1.71 (0.22–5.1)	5.72 ± 1.59 (3.52–7.51)^a^	1.81 ± 0.18 (1.63–2.11)^b^	1.84 ± 0.68 (1.22–3.14)^b^	**0.006**
MDA (*μ*mol/L)	4.06 ± 0.58 (3.43–5.01)	8.68 ± 1.16 (6.45–9.87)^a^	4.59 ± 1.92 (3.07–8.29)^b^	4.73 ± 1.71 (3.22–8.08)^b^	**0.006**
Protein carbonyl (nmol/mL)	1403.6 ± 397.4 (1073.5–2109.8)	2358.2 ± 190.1 (2071.6–2597.1)^a^	1688.4 ± 204.6 (1453.1–2053.1)^b^	1826.9 ± 317.7 (1389.8–2214.6)^b^	**0.003**

Data were shown as mean ± standard deviation and range (min–max).

^a^There was statistically significant difference from group 1.

^b^There was statistically significant difference from group 2.

**Table 4:** Johnsen Scores and the statistical analysis results.

	Group 1 (*n* = 7)	Group 2 (*n* = 7)	Group 3 (*n* = 7)	Group 4 (*n* = 7)	*P*
Johnsen Score	9.05 ± 0.37 (8.7–9.6)	8.68 ± 0.89 (7.4–9.6)	8.83 ± 0.37 (8.3–9.2)	9.1 ± 0.39 (8.6–9.7)	0.727

Data were shown as mean ± standard deviation and range (min–max).
